# Five-Minute Oxygen Saturation and Delivery Room Oxygen Requirements as Early Markers of Illness Severity in Preterm Infants ≤32 Weeks of Gestation: A Single-Centre Retrospective Cohort Study

**DOI:** 10.3390/medicina62071426

**Published:** 2026-07-22

**Authors:** Ema Šlabek, Koraljka Manestar Rukavina, Dorotea Drašković, Maja Zaninović, Ana Milardović, Maja Ješić, Lucija Matko, Iva Bilić Čače

**Affiliations:** 1 Department of Pediatrics, University Hospital Centre Rijeka, 51000 Rijeka, Croatia; ema.slabek@medri.uniri.hr (E.Š.); dorotea.draskovic@medri.uniri.hr (D.D.); maja.zaninovic@medri.uniri.hr (M.Z.); ana.milardovic@medri.uniri.hr (A.M.); maja.jesic@medri.uniri.hr (M.J.); lucija.matko@medri.uniri.hr (L.M.); iva.bilic@medri.uniri.hr (I.B.Č.); 2 Faculty of Medicine, University of Rijeka, 51000 Rijeka, Croatia; 3 Division of Neonatal and Pediatric Intensive Care, Department of Pediatrics, University Hospital Centre Rijeka, 51000 Rijeka, Croatia

**Keywords:** oxygen saturation, infant, premature, diseases, cerebral intraventricular hemorrhage, noninvasive ventilation

## Abstract

*Background and objectives*: Optimal oxygen administration during delivery room resuscitation remains a major challenge in very preterm infants. While both hypoxemia and excessive oxygen exposure have been associated with adverse outcomes, the prognostic value of early oxygenation parameters remains unclear. This study aimed to evaluate the association between preductal SpO_2_ at 5 min of life and early respiratory and neurological outcomes in preterm infants born at or below 32 weeks of gestation. *Materials* *and* *methods*: This retrospective single-centre study included 31 preterm infants born at ≤32 weeks of gestation, identified from 34 consecutive eligible births. Demographic, delivery room, respiratory, laboratory, and neuroimaging data were collected from medical records. The primary outcome was intraventricular hemorrhage (IVH), graded by the Papile classification, during the first week of life. Secondary outcomes included respiratory support requirements, surfactant administration, and capillary blood gas parameters at two hours of life. Associations were evaluated using Spearman rank correlation; because SpO_2_@5 and delivery room FiO_2_ were strongly related to gestational age, partial correlations controlling for gestational age were additionally performed, together with a group comparison for IVH. Exact two-sided *p* values are reported. *Results*: The median gestational age was 28 weeks + 4 days (range: 22 weeks + 1 day to 31 weeks + 5 days), and the median birth weight was 1120 g. IVH of any grade occurred in 20 infants (64.5%), including severe (grade III–IV) IVH in 10 (32.3%). Median delivery room FiO_2_ was 35% (IQR 30–50%), and median SpO_2_ at 5 min of life was 83% (IQR 75–90%). Lower SpO_2_ at 5 min of life was associated with an increased number of surfactant doses (rs = −0.46, *p* = 0.010), lower capillary pH at two hours of life (rs = 0.39, *p* = 0.032), and a higher grade of IVH (rs = −0.39, *p* = 0.030); median SpO_2_@5 was 80% in infants with IVH versus 90% in those without (*p* = 0.057). Higher delivery room FiO_2_ was associated with increased use of DuoPAP (rs = 0.36, *p* = 0.045), mechanical ventilation (rs = 0.41, *p* = 0.021), and surfactant administration (rs = 0.56, *p* < 0.001), as well as lower pH (rs = −0.55, *p* = 0.002) and higher lactate levels (rs = 0.37, *p* = 0.044) at two hours of life. Because SpO_2_@5 and FiO_2_ were both strongly correlated with gestational age (rs = 0.54 and −0.76, respectively), all associations were re-examined controlling for gestational age. The associations of SpO_2_@5 with IVH grade (partial rs = −0.22, *p* = 0.247), surfactant doses (partial rs = −0.08, *p* = 0.678) and 2 h pH (partial rs = 0.19, *p* = 0.335) were attenuated and no longer significant, as were those of FiO_2_ with mechanical ventilation (partial rs = −0.04, *p* = 0.817) and surfactant doses (partial rs = −0.01, *p* = 0.945); only the association between FiO_2_ and 2 h pH persisted (partial rs = −0.37, *p* = 0.048). *Conclusions*: In preterm infants born at or below 32 weeks of gestation, lower SpO_2_ at 5 min and higher delivery room oxygen requirements were associated with less favorable respiratory and metabolic outcomes, as well as with IVH. However, apart from the association between FiO_2_ and 2 h pH, none of these associations remained statistically significant after adjustment for gestational age, and these findings, from a small and heterogeneous single-centre cohort, should be considered hypothesis-generating. Early oxygenation parameters are best viewed as simple bedside markers of cardiopulmonary adaptation and overall illness severity in very preterm infants, rather than as independent predictors of clinical outcomes. Further prospective studies are needed to confirm these associations and define clinically relevant threshold values.

## 1. Introduction

Preterm infants born at or below 32 weeks of gestation face a challenging transition from fetal to extrauterine life. Most require respiratory support and supplemental oxygen immediately after birth because of structural and functional immaturity of the lungs [1]. They are particularly vulnerable to oxidative stress due to immature antioxidant defense systems [2]. Consequently, the same oxygen that facilitates successful cardiopulmonary adaptation may also contribute to oxidative injury. This oxidative stress is a primary pathogenic factor in major neonatal morbidities, including bronchopulmonary dysplasia, retinopathy of prematurity, and intraventricular hemorrhage (IVH) [1]. Therefore, oxygen administration in the delivery room requires a careful balance between avoiding hypoxia and minimizing oxygen toxicity.

While there is a global consensus on the need for cautious oxygen administration, finding the “ideal” starting point for the initial FiO_2_ remains one of the most debated topics in neonatal medicine [3]. Current international resuscitation guidelines generally recommend initiating respiratory support with lower oxygen concentrations and titrating oxygen according to preductal saturation targets. However, recent European Consensus Guidelines for Respiratory Distress Syndrome suggest that infants born before 29 weeks of gestation may benefit from an initial FiO_2_ of 0.60 at birth rather than 0.30, primarily to reduce severe bradycardia and the need for advanced resuscitation [4]. This illustrates the ongoing uncertainty regarding the optimal initial oxygen strategy in extremely preterm infants. From this starting point, clinicians must titrate oxygen carefully, adjusting FiO_2_ according to preductal SpO_2_ targets, with the primary goal of reaching an SpO_2_ of 80% by the 5th minute of life [5]. This balance is difficult to strike: a strategy that is too conservative may lead to prolonged hypoxemia and bradycardia, while a more aggressive approach risks triggering irreversible oxygen-mediated injury [1,6]. To address this uncertainty, large-scale randomized trials such as the High or Low Initial Oxygen Trial (HiLo) study are currently evaluating whether a lower (30%) or higher (60%) initial FiO_2_ provides a safer pathway to better neurodevelopmental and clinical outcomes [6].

Given the ongoing uncertainty regarding the most effective oxygen strategies, clinical focus has increasingly shifted toward identifying early, reliable physiological markers of a successful transition. Preductal SpO_2_ at 5 min of life (SpO_2_@5) has emerged as a particularly valuable metric; it reflects the interplay among pulmonary aeration, cardiovascular adaptation, and the infant’s response to respiratory support and oxygen supplementation [5]. Achieving an SpO_2_@5 above 80% is now recognized as a critical clinical milestone. Research has shown that infants who fail to achieve this target are at increased risk of mortality and severe neurological complications, including IVH [7]. However, less is known about how these early saturation levels relate to the respiratory challenges and neurological outcomes encountered in routine clinical practice [3].

This study aimed to evaluate the association between SpO_2_@5 and early respiratory and neurological outcomes in preterm infants born at or below 32 weeks of gestation. Specifically, we investigated the relationship between SpO_2_@5, respiratory support requirements and the occurrence of IVH during the first week of life. We additionally explored associations between delivery room oxygen exposure, early acid–base status, and short-term clinical outcomes. Although the prognostic value of a low SpO_2_@5 has been described mainly in relation to mortality and severe IVH, we also examined how SpO_2_@5 and delivery room FiO_2_ relate, within the same cohort, to the day-to-day respiratory support requirements of the first week of life and to early acid–base status in routine clinical practice.

## 2. Materials and Methods

This retrospective cohort study was conducted at the Department of Paediatrics, Clinical Hospital Centre Rijeka, Croatia, a tertiary perinatal referral centre. Medical records of preterm infants born at or below 32 weeks of gestation and admitted to the neonatal intensive care unit were reviewed. The study was approved by the institutional Ethics Committee, and informed consent was obtained. 

**Figure 1:**
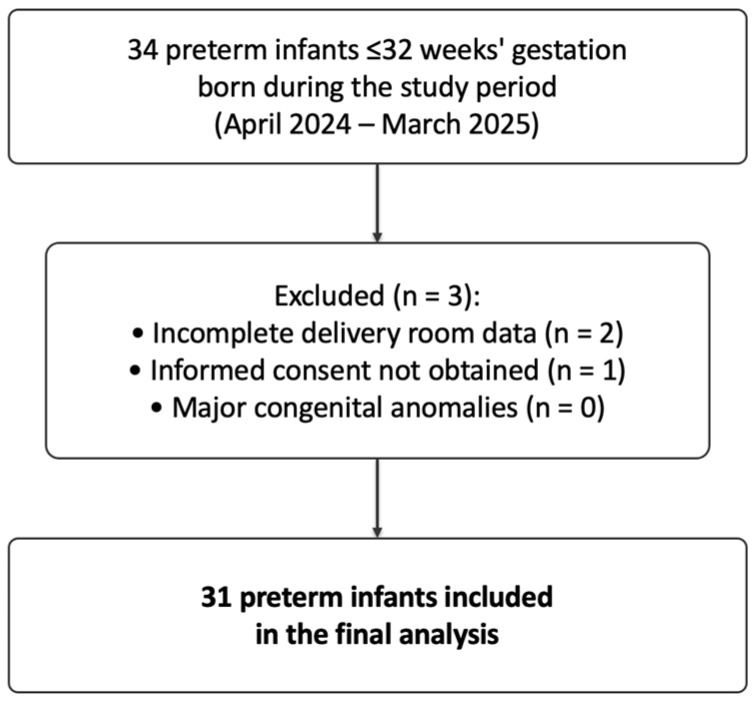
Participant flow diagram. Of 34 eligible preterm infants ≤32 weeks’ gestation born during the study period, 3 were excluded (2 for incomplete delivery room data, 1 for lack of informed consent; no congenital anomalies occurred), leaving 31 infants for analysis.

All preterm infants born at or below 32 weeks of gestation during a 12-month study period (April 2024–March 2025) were eligible for inclusion. Infants with major congenital anomalies known to affect cardiopulmonary transition, as well as those with incomplete delivery room data, were excluded. Of the 34 eligible infants, two were excluded for incomplete delivery room data and one because informed consent was not obtained; no infant had a major congenital anomaly during the study period. The final study cohort consisted of 31 infants (Figure 1). 

Demographic and perinatal variables included gestational age, birth weight, sex, mode of delivery, time of birth, antenatal corticosteroid exposure, and Apgar scores at 1, 5, and 10 min. Delivery room variables comprised FiO_2_, use of positive end-expiratory pressure and positive-pressure ventilation, SpO_2_@5, and umbilical cord blood gas parameters (pH, base excess, and lactate). Preductal oxygen saturation was monitored with an EDAN H100 handheld pulse oximeter (EDAN Instruments Co., Ltd., Shenzhen, China; Nellcor OxiMax signal processing) and a single-use Nellcor MAX-N-I neonatal sensor (Medtronic, Minneapolis, MN, USA) applied to the right hand. At the 5 min mark, the displayed value was read and recorded by a dedicated team member once the plethysmographic waveform and displayed pulse rate were stable and consistent with the infant’s auscultated heart rate. This reading is a short-term average computed by the device rather than an instantaneous value, with an averaging window that is fixed by the manufacturer and not user-set as a specific number of seconds. Respiratory outcomes recorded during the first week of life included the use and duration of nasal continuous positive airway pressure (nCPAP), dual-level positive airway pressure (DuoPAP), high-flow nasal cannula (HFNC) support, and mechanical ventilation (MV). These modalities were not mutually exclusive: infants could receive more than one form of support sequentially during the first week, and 17 infants received both nCPAP and DuoPAP. Each modality was therefore analysed separately as a binary variable (ever used during the first week of life); no composite non-invasive support variable was used, and the duration of each modality is reported descriptively among the infants who received it. Surfactant administration, including the number of doses and time to first administration, was also documented. Laboratory variables included capillary blood gas measurements obtained at two hours of life, including pH, bicarbonate concentration, partial pressure of carbon dioxide, and lactate levels. Neuroimaging data were obtained from cranial ultrasound examinations performed routinely in all infants during the first week of life and again before discharge, per unit protocol, with additional scans when clinically indicated. IVH was graded according to the Papile classification, and the highest grade documented during the first week of life was used for analysis. 

The primary outcome was the occurrence of IVH (any Papile grade) detected on cranial ultrasound during the first week of life. Secondary outcomes included the requirement for MV, nCPAP and DuoPAP support, surfactant administration, and capillary pH and lactate values at two hours of life. Given the retrospective design, no statistical analysis plan was registered in advance; analyses treating IVH grade as an ordinal variable, the gestational-age-adjusted partial correlations, and the effect estimates for low SpO_2_@5 were exploratory and are reported as such.

Statistical analyses were performed using MedCalc Statistical Software version 23.1.3 (MedCalc Software Ltd., Mariakerke, Belgium). Continuous variables are presented as median and interquartile range (IQR), whereas categorical variables are reported as absolute and relative frequencies. Categorical variables were compared using the chi-square test. Normality of distribution was assessed using the Kolmogorov–Smirnov test. Associations between variables were evaluated using Spearman rank correlation coefficients (rs), with exact two-sided *p* values reported. Because both SpO_2_@5 and delivery room FiO_2_ were strongly correlated with gestational age, partial Spearman correlations controlling for gestational age were additionally computed. SpO_2_@5 was compared between infants with and without IVH using the Mann–Whitney U test, and the association between low SpO_2_@5 (<80%) and the occurrence of IVH was summarised as a crude (unadjusted) odds ratio (OR) and a crude relative risk (RR) with 95% confidence intervals (CIs), derived from the 2 × 2 table presented in Section 3.4, with Fisher’s exact test used for this comparison. A two-sided *p* value < 0.05 was considered statistically significant. Given the small sample size, the wide range of gestational ages, and the absence of correction for multiple comparisons, all findings should be interpreted as hypothesis-generating.

## 3. Results

### 3.1. Baseline Characteristics

Of 34 eligible infants, 31 were included in the analysis (Figure 1). The median gestational age was 28 + 4 weeks (range 22 + 1–31 + 5), and the median birth weight was 1120 g (IQR 937.5–1460 g). Nineteen infants (61.3%) were male. Median delivery room FiO_2_ was 35% (IQR 30–50%), and median SpO_2_@5 was 83% (IQR 75–90%). Baseline demographic and delivery room characteristics are summarized in Table 1.

### 3.2. Clinical Outcomes and Event Frequencies

IVH of any grade was detected on first-week cranial ultrasound in 20 infants (64.5%): grade I in 4 (12.9%), grade II in 6 (19.4%), grade III in 5 (16.1%), and grade IV in 5 (16.1%); severe IVH (grade III–IV) occurred in 10 (32.3%). At least one dose of surfactant was given to 21 infants (67.7%), MV was required in 15 (48.4%), and non-invasive respiratory support was used in most infants. Five infants (16.1%) died within 7 days and two more (6.5%) between days 8 and 28 (28-day mortality 7/31, 22.6%). Event frequencies and early physiological parameters are summarized in Table 2.

### 3.3. Associations Between SpO_2_@5 and Early Respiratory and Neurological Outcomes

Higher SpO_2_@5 was associated with more favorable early clinical outcomes. SpO_2_@5 correlated positively with gestational age (rs = 0.54, *p* = 0.002) and capillary pH at two hours of life (rs = 0.39, *p* = 0.032), and negatively with the number of surfactant doses administered (rs = −0.46, *p* = 0.010). Lower SpO_2_@5 was also associated with a higher grade of IVH during the first week of life (rs = −0.39, *p* = 0.030). Median SpO_2_@5 was 80% (IQR 74–88) in infants who developed IVH versus 90% (IQR 84–92) in those who did not (*p* = 0.057). Among infants with SpO_2_@5 below 80%, 8 of 10 (80.0%) developed IVH, compared with 12 of 21 (57.1%) infants with SpO_2_@5 ≥80%; this corresponds to a crude OR of 2.58 (95% CI 0.50–13.36) and a crude RR of 1.40 (95% CI 0.86–2.27; Fisher’s exact *p* = 0.262). After controlling for gestational age, the associations of SpO_2_@5 with IVH grade (partial rs = −0.22, *p* = 0.247), the number of surfactant doses (partial rs = −0.08, *p* = 0.678) and capillary pH at two hours of life (partial rs = 0.19, *p* = 0.335) were attenuated and no longer statistically significant. Unadjusted and gestational-age-adjusted correlations, with exact *p* values and the number of infants included in each analysis, are summarized in Table 3.

### 3.4. Associations Between Delivery Room FiO_2_ and Early Respiratory and Biochemical Parameters

Higher delivery room FiO_2_ was associated with lower gestational age (rs = −0.76, *p* < 0.001) and a greater need for respiratory support during the first week of life. Positive correlations were observed between FiO_2_ and DuoPAP use (rs = 0.36, *p* = 0.045), MV (rs = 0.41, *p* = 0.021), and the number of surfactant doses administered (rs = 0.56, *p* < 0.001), whereas the correlation between FiO_2_ and nCPAP use was not significant (rs = −0.26, *p* = 0.159). Higher FiO_2_ was also associated with lower capillary pH (rs = −0.55, *p* = 0.002), lower bicarbonate concentrations (rs = −0.50, *p* = 0.005), and higher lactate levels (rs = 0.37, *p* = 0.044) at two hours of life. After controlling for gestational age, the associations of FiO_2_ with DuoPAP use (partial rs = 0.19, *p* = 0.302), MV (partial rs = −0.04, *p* = 0.817), the number of surfactant doses (partial rs = −0.01, *p* = 0.945), bicarbonate (partial rs = −0.24, *p* = 0.204) and lactate (partial rs = 0.06, *p* = 0.761) were attenuated and no longer statistically significant; only the association with capillary pH at two hours of life persisted (partial rs = −0.37, *p* = 0.048). Unadjusted and gestational-age-adjusted correlations, with exact *p* values and the number of infants included in each analysis, are summarized in Table 4 and Table 5.

Crude odds ratio 2.58 (95% CI 0.50–13.36); crude relative risk 1.40 (95% CI 0.86–2.27); Fisher’s exact *p* = 0.262. Estimates are unadjusted; the association between SpO_2_@5 and IVH was no longer statistically significant after adjustment for gestational age (Table 3).

## 4. Discussion

### 4.1. Principal Findings

In this retrospective cohort study of preterm infants born at or below 32 weeks of gestation, we observed associations between oxygenation during the immediate postnatal period and early respiratory and neurological outcomes. Lower SpO_2_@5 was associated with a higher grade of IVH (rs = −0.39, *p* = 0.030), consistent with the recognised sensitivity of the preterm brain during the fragile transition to extrauterine life [7]. Beyond neurological outcomes, lower SpO_2_@5 was associated with increased surfactant requirements (rs = −0.46, *p* = 0.010) and lower pH at two hours of life (rs = 0.39, *p* = 0.032). Because both parameters were strongly correlated with gestational age, these associations were attenuated after adjustment and are best interpreted as reflecting the degree of prematurity and overall illness severity rather than an independent effect of early oxygenation.

We also observed that higher delivery room FiO_2_ was associated with increased respiratory support requirements, including MV (rs = 0.41, *p* = 0.021), DuoPAP use (rs = 0.36, *p* = 0.045), and surfactant administration (rs = 0.56, *p* < 0.001) during the first week of life.

Collectively, these observations are consistent with a relationship between early oxygenation and cardiopulmonary adaptation. Because none of these associations was independent of gestational age, SpO_2_@5 and delivery room FiO_2_ are best regarded as readily available markers of the degree of prematurity and overall illness severity, rather than as independent prognostic indicators.

### 4.2. SpO_2_@5 and Respiratory Adaptation

Our findings support the growing recognition of SpO_2_@5 as an integrative marker of successful neonatal transition. In the present study, lower SpO_2_@5 was associated with increased surfactant requirements (rs = −0.46, *p* = 0.010) and less favorable acid–base status, including lower pH at two hours of life (rs = 0.39, *p* = 0.032). These results indicate that SpO_2_@5 reflects not only the effectiveness of respiratory stabilization but also the overall quality of cardiopulmonary adaptation in very preterm infants. This is biologically plausible, as SpO_2_ during the first minutes of life reflects the effectiveness of lung aeration, establishment of functional residual capacity, pulmonary blood flow, and gas exchange [8]. Failure to achieve adequate oxygenation may indicate more severe respiratory distress syndrome and impaired pulmonary transition, both of which increase the likelihood of surfactant administration [9]. Interestingly, both variables incorporated into the Oxygen Saturation Index (OSI), namely FiO_2_ and SpO_2_, were significantly associated with adverse respiratory outcomes in our cohort, including surfactant administration (SpO_2_: rs = −0.46, *p* = 0.010; FiO_2_: rs = 0.56, *p* < 0.001). Although OSI was not calculated in the present study, recent investigations have suggested that composite oxygenation indices may provide useful non-invasive markers of respiratory disease severity and surfactant requirement in preterm infants [9,10,11].

The observed relationship between lower SpO_2_@5, lower pH, and higher lactate concentrations at two hours of life further supports the link between suboptimal early oxygenation and impaired metabolic adaptation during the immediate postnatal period. Inadequate oxygenation during neonatal transition may compromise tissue oxygen delivery, resulting in anaerobic metabolism, lactate accumulation, and metabolic acidosis [12]. Hypoxemia and acidosis may further increase pulmonary vascular resistance and impair pulmonary blood flow, creating a vicious cycle that compromises oxygenation and cardiopulmonary adaptation [13]. Taken together, these findings support the role of SpO_2_@5 as an integrative marker of early cardiopulmonary adaptation and disease severity in very preterm infants.

### 4.3. SpO_2_@5 and IVH

A clinically relevant observation was the association between lower SpO_2_@5 and IVH during the first week of life (rs = −0.39, *p* = 0.030), with a median SpO_2_@5 of 80% in infants with IVH versus 90% in those without. Increasing evidence suggests that SpO_2_@5 is not only a marker of successful respiratory transition but also an indicator of neurological vulnerability in very preterm infants. Previous studies have shown that failure to achieve an SpO_2_ of 80% by 5 min of life is associated with an increased risk of severe IVH and mortality [7,8]. Infants with SpO_2_@5 below 80% had a crude OR for IVH of 2.58 (95% CI 0.50–13.36) and a crude RR of 1.40 (95% CI 0.86–2.27); with few events, these estimates are imprecise and their confidence intervals include unity. The association between SpO_2_@5 and IVH did not remain statistically significant after adjustment for gestational age (partial rs = −0.22, *p* = 0.247). This finding should therefore be regarded as hypothesis-generating and does not establish SpO_2_@5 as an independent marker of neurological risk.

Several pathophysiological mechanisms may explain this relationship. The immature germinal matrix vasculature of very preterm infants is highly susceptible to injury, particularly during periods of hemodynamic instability [3]. Inadequate oxygenation during neonatal transition may result in cerebral hypoxia, compensatory cerebral vasodilation, and impaired autoregulation of cerebral blood flow, thereby increasing the vulnerability of fragile germinal matrix vessels to rupture [14]. Recent near-infrared spectroscopy studies have further highlighted the importance of stable cerebral oxygenation during the immediate postnatal period, demonstrating an association between disturbances in cerebral oxygen delivery and brain injury in extremely preterm infants [14,15]. Hypoxemia-associated metabolic acidosis and subsequent reoxygenation may contribute to oxidative endothelial injury and fluctuations in cerebral perfusion, further increasing the susceptibility of the immature cerebral vasculature to hemorrhage [14]. Collectively, these mechanisms provide biological plausibility for an association between early oxygenation and cerebral injury. In our cohort, however, this association was not independent of gestational age, and our data therefore do not support the use of SpO_2_@5 as a stand-alone bedside assessment of neurological vulnerability.

### 4.4. Delivery Room FiO_2_ and Early Respiratory Outcomes

In this study, higher delivery room FiO_2_ was associated with increased use of DuoPAP (rs = 0.36, *p* = 0.045), MV (rs = 0.41, *p* = 0.021), and surfactant administration (rs = 0.56, *p* < 0.001) during the first week of life. The strongest correlation was observed between FiO_2_ and gestational age (rs = −0.76, *p* < 0.001), with the most immature infants requiring the highest levels of oxygen supplementation. Oxygen requirements during neonatal stabilization reflect both the degree of prematurity and the severity of early respiratory compromise.

Oxygen exposure during stabilization may provide important information regarding the adequacy of pulmonary transition. Recent European Consensus Guidelines for Respiratory Distress Syndrome continue to recommend consideration of surfactant therapy when FiO_2_ exceeds approximately 0.30 despite CPAP support, while emerging evidence suggests that CPAP failure may be predicted at even lower oxygen thresholds [4]. Furthermore, lung ultrasound has emerged as a valuable tool for identifying surfactant deficiency, demonstrating greater sensitivity than FiO_2_ thresholds alone for predicting surfactant need [16]. In our cohort, higher delivery room FiO_2_ was associated with both increased surfactant administration and greater respiratory support requirements, supporting its role as an early marker of respiratory disease severity.

Notably, recommendations regarding initial oxygen administration continue to evolve. The most recent European Consensus Guidelines suggest initiating stabilization with FiO_2_ 0.60 in infants born before 29 weeks of gestation, reflecting growing recognition that extremely preterm infants may require higher initial oxygen concentrations to achieve adequate oxygenation and avoid severe hypoxemia during transition [4].

In our cohort, higher delivery room FiO_2_ was associated with lower capillary pH, higher lactate, and lower bicarbonate concentrations at two hours of life (rs = −0.50, *p* = 0.005). This pattern is physiologically coherent and consistent with a more pronounced metabolic acidosis in the most immature and most compromised infants.

Capillary blood gas values at two hours of life are influenced by ventilation, oxygen therapy, surfactant, and fluids, and therefore reflect early treatment as well as perinatal insult; umbilical cord blood gas parameters and Apgar scores describe the perinatal condition more directly. In our cohort, cord blood gas data were available only for a subset of infants (pH in 22, base excess in 12, and lactate in 14), which limits their interpretation. The relationship between Apgar score and umbilical cord acid–base status is itself only weak-to-moderate [17].

Our findings also need to be seen against the limited prognostic value of single, routinely available perinatal markers. A statistically significant association between such a marker and a neonatal outcome does not necessarily translate into clinically useful prediction for an individual infant: first-trimester screening markers, for example, show statistically significant but weak relationships with Apgar score, umbilical cord pH and admission to neonatal intensive care, with prognostic areas under the curve below 0.7 [18]. The same caution applies to SpO_2_@5 and delivery room FiO_2_ in our cohort, where the associations with early outcomes operated largely through their relationship with gestational age and none were independent of it.

### 4.5. Limitations

This study has several limitations. Its retrospective single-centre design and relatively small sample size limited statistical power and precluded formal multivariable adjustment for confounding factors, particularly gestational age; in exploratory partial-correlation analyses, most associations were attenuated after accounting for gestational age, indicating that they are substantially influenced by the degree of prematurity. The cohort also spanned a very wide range of gestational ages (22 + 1 to 31 + 5 weeks), with markedly different baseline risks, and the sample size did not allow identification of clinically relevant threshold values, robust analysis of severe IVH, or correction for multiple comparisons. In addition, the small number of events precluded formal IVH-grade-stratified analysis. Finally, only short-term outcomes were evaluated, and long-term neurodevelopmental follow-up was not available. Umbilical cord blood gas data were available for only a subset of infants. Nevertheless, detailed delivery room data, including both FiO_2_ and SpO_2_@5 and complete first-week IVH grading, were available for all infants, enabling assessment of the relationship between early oxygenation and subsequent respiratory and neurological outcomes.

### 4.6. Clinical Implications

No clinically relevant threshold values could be established and, because the observed associations were not independent of gestational age, our data do not support the use of SpO_2_@5 or delivery room FiO_2_ as stand-alone tools to identify infants at increased risk of neurological complications. A low saturation and a high oxygen requirement during the first minutes after birth are best interpreted as part of the overall clinical picture of a very immature and unwell infant. Independently of these findings, careful oxygen titration during neonatal stabilization remains important, with the goal of achieving recommended saturation targets while minimizing both hypoxemia and excessive oxygen exposure.

## 5. Conclusions

In preterm infants born at or below 32 weeks of gestation, lower SpO_2_@5 and higher delivery room FiO_2_ were associated with less favorable respiratory and metabolic outcomes and with IVH. Because these associations were not independent of gestational age and derive from a small, heterogeneous cohort, early oxygenation parameters are best regarded as simple bedside markers that integrate the degree of prematurity and overall disease severity rather than as independent predictors. Further prospective studies with multivariable adjustment are needed to confirm these associations and define clinically relevant threshold values.

## Figures and Tables

**Table 1:** Baseline demographic and delivery room characteristics of preterm infants ≤32 weeks’ gestation (*N* = 31).

Characteristic	Value
Male sex, *n* (%)	19 (61.3)
Gestational age, weeks, median (range)	28 + 4 (22 + 1–31 + 5)
Birth weight, g, median (IQR)	1120 (937.5–1460)
Delivery room FiO_2_, %, median (IQR)	35 (30–50)
Preductal oxygen saturation at 5 min, %, median (IQR)	83 (75–90)
PEEP applied, *n* (%)	27 (87.1)
PPV applied, *n* (%)	14 (45.2)

**Table 2:** Clinical outcomes, event frequencies, and early physiological parameters (*N* = 31).

Variable	Value
IVH, any grade, *n* (%)	20 (64.5)
Grade I/II/III/IV, *n*	4/6/5/5
Severe IVH (grade III–IV), *n* (%)	10 (32.3)
Surfactant, ≥1 dose, *n* (%)	21 (67.7)
1/2/3 doses, *n*	15/4/2
Mechanical ventilation, *n* (%)	15 (48.4)
nCPAP/DuoPAP/HFNC, *n* (%)	20 (64.5)/24 (77.4)/12 (38.7)
Delivery room PEEP/PPV, *n* (%)	27 (87.1)/14 (45.2)
Death ≤ 7 d/8–28 d, *n* (%)	5 (16.1)/2 (6.5)
Apgar at 1/5 min, median	6 (4–7)/7 (6–8)
Umbilical cord pH, median (IQR) [*n* = 22]	7.29 (7.21–7.34)
Umbilical cord base excess, median (IQR) [*n* = 12]	−3.0 (−3.7 to −1.1)
Umbilical cord lactate, median (IQR) [*n* = 14]	2.5 (2.1–3.3)
Capillary pH at 2 h, median (IQR)	7.30 (7.26–7.34)
Capillary pCO_2_/HCO_3_ at 2 h, median	6.5 kPa/24.4 mmol/L
Capillary lactate at 2 h, mmol/L, median (IQR)	1.6 (1.2–1.9)
nCPAP duration, h, median (IQR) [*n* = 20]	20 (5–57)
DuoPAP duration, h, median (IQR) [*n* = 23]	5 (3–34)
HFNC duration, h, median (IQR) [*n* = 12]	42 (12–78)
Mechanical ventilation duration, h, median (IQR) [*n* = 15]	10 (6–171)

Values are *n* (%) unless otherwise stated. Cord blood gas parameters were available for a subset of infants, as indicated. IVH, intraventricular hemorrhage; HFNC, high-flow nasal cannula; PEEP, positive end-expiratory pressure; PPV, positive-pressure ventilation.

**Table 3:** Correlations between SpO_2_ at 5 min of life and early clinical outcomes.

Variable	*n*	Unadjusted rs	*p*	Adjusted rs (For GA)	*p*
Gestational age	31	0.54	0.002	—	—
Capillary pH at 2 h of life	30	0.39	0.032	0.19	0.335
Number of surfactant doses	31	−0.46	0.010	−0.08	0.678
IVH grade during the first week of life	31	−0.39	0.030	−0.22	0.247

*n*, number of infants included in the analysis; rs, Spearman rank correlation coefficient; adjusted rs, partial Spearman correlation controlling for gestational age (GA). Em dash (—) indicates not applicable. Exact two-sided *p* values are reported.

**Table 4:** Correlations between delivery room FiO_2_ and early respiratory and biochemical outcomes.

Variable	*n*	Unadjusted rs	*p*	Adjusted rs (for GA)	*p*
Gestational age	31	−0.76	<0.001	—	—
nCPAP use	31	−0.26	0.159	−0.01	0.941
DuoPAP use	31	0.36	0.045	0.19	0.302
Mechanical ventilation	31	0.41	0.021	−0.04	0.817
Number of surfactant doses	31	0.56	<0.001	−0.01	0.945
Capillary pH at 2 h of life	30	−0.55	0.002	−0.37	0.048
Capillary bicarbonate concentration at 2 h of life	31	−0.50	0.005	−0.24	0.204
Capillary lactate concentration at 2 h of life	30	0.37	0.044	0.06	0.761

*n*, number of infants included in the analysis; rs, Spearman rank correlation coefficient; adjusted rs, partial Spearman correlation controlling for gestational age (GA); nCPAP, nasal continuous positive airway pressure; DuoPAP, dual-level positive airway pressure. Em dash (—) indicates not applicable. Exact two-sided *p* values are reported.

**Table 5:** Two-by-two table of preductal SpO_2_ at 5 min of life (<80% vs. ≥80%) and the occurrence of intraventricular hemorrhage during the first week of life (*N* = 31).

SpO_2_@5	IVH Present, *n*	IVH Absent, *n*	Total, *n*
<80%	8	2	10
≥80%	12	9	21
Total	20	11	31

## Data Availability

The data presented in this study are available from the corresponding author upon reasonable request. The data are not publicly available due to privacy and ethical restrictions.

## Abbreviations

The following abbreviations are used in this manuscript:

FiO_2_	Fraction of inspired oxygen
PEEP	Positive end-expiratory pressure
PPV	Positive pressure ventilation
IQR	Interquartile range
SpO_2_@5	Preductal oxygen saturation at 5 min of life
rs	Spearman rank correlation coefficient. Positive rs values indicate direct correlations, whereas negative rs values indicate inverse correlations
nCPAP	Nasal continuous positive airway pressure
IVH	Intraventricular hemorrhage
MV	Mechanical ventilation
CPAP	Continuous positive airway pressure
HFNC	High-flow nasal cannula
RDS	Respiratory distress syndrome
OSI	Oxygen Saturation Index
NIRS	Near-infrared spectroscopy
OR	Odds ratio
RR	Relative risk
CI	Confidence interval
DuoPAP	Dual-level (bilevel) positive airway pressure
